# Vitamin D Supplementation in Critically Ill—Narrative Review

**DOI:** 10.3390/nu17010156

**Published:** 2024-12-31

**Authors:** Lenko Saric, Josipa Domazet Bugarin, Svjetlana Dosenovic

**Affiliations:** 1 Department of Anesthesiology, Reanimatology and Intensive Care, University Hospital Split, 21000 Split, Croatia; jdomazet@kbsplit.hr (J.D.B.); sdosenovic@kbsplit.hr (S.D.); 2 University Department of Health Studies, University of Split, 21000 Split, Croatia

**Keywords:** vitamin D, supplementation, critically ill, ICU

## Abstract

Background: Studies have shown a high prevalence of vitamin D deficiency in critically ill patients, and these patients are at higher risk for pneumonia and have increased incidence of sepsis and mortality. In this study, we reviewed available literature from randomized controlled trials (RCTs) on vitamin D supplementation in critically ill patients and summarized the evidence in this narrative review. Methods: Randomized controlled trials that included vitamin D supplementation as an intervention were eligible for inclusion. No limits were set regarding vitamin D dosage or route of administration, as well as for primary and secondary outcomes. A search was conducted in MEDLINE via PubMed for eligible RCTs. References from systematic reviews (SRs) and meta-analyses (MAs) were screened, and Clinicaltrials.gov was searched for ongoing studies. Results: A total of 21 RCTs involving 3166 patients were analyzed. There was a large heterogeneity in terms of patients’ characteristics and inclusion criteria. Only six studies included patients with vitamin D levels < 50 nmol/L. Regarding clinically important outcomes, most of the studies did not show differences between the intervention and control group in terms of mortality, intensive care unit (ICU) or hospital length of stay (LoS). Conclusions: There is great variability in trial designs regarding the selection of patients, dosage, dosing intervals and routes of administration of vitamin D supplements. Better study designs are mandatory for future clinical research, with measuring and reporting basal vitamin D levels before randomization. Since variability in supplementation regimes limits the possibility of data synthesis, standardized protocols for vitamin D supplementation should be used in clinical trial settings.

## 1. Introduction

Lee et al. have found a high prevalence of vitamin D deficiency in critically ill adult and pediatric patients [1], and these findings were confirmed in a number of studies [2,3,4]. Furthermore, in critically ill patients, vitamin D metabolism is often dysregulated, which can further lower blood levels [5]. Low vitamin D levels have been associated with an increased incidence of sepsis, acute respiratory distress as well as acute kidney injury [6,7,8]. It has also been shown that patients with vitamin D deficiency have higher mortality rates and longer ICU stays [4].

Vitamin D is a hormone primarily involved in the homeostasis of calcium and phosphate [9]. In addition, it is known to have an immunomodulatory effect, especially in respiratory tract infections [10]. It exerts its action by modulating both the innate and adaptive immune responses [11]. Activated immune cells synthesize 1,25 dihydrocholecalciferol and regulate the production of antimicrobial proteins involved in immune responses [12,13,14]. Furthermore, it acts as an anti-inflammatory agent [13,14]. Other than immunomodulatory effects, vitamin D has an effect on calcium homeostasis and bone metabolism [15,16]. Furthermore, it affects the musculoskeletal system by reducing muscle wasting, and it has a possible effect on cardiac function [17,18,19,20].

There is evidence that vitamin D supplementation protects against respiratory tract infections, including a possible protective role against acute lung injury (ARDS) [21,22]. However, while some studies have found a reduction in disease severity and earlier recovery with vitamin D supplementation, others have shown no difference in outcomes [23,24,25,26,27,28,29,30,31].

All of these systems are often affected in critically ill patients and contribute to longer ICU and hospital LoS as well as prolonged rehabilitation of these patients. Therefore, it is reasonable to assume that supplementation of vitamin D could bring clinical benefit to critically ill patients.

Guidelines relating to the general population clearly define normal vitamin D values as well as the minimal daily requirements for vitamin D3 [32]. Minimal requirements for vitamin D3 are also defined for specific populations who are at risk of deficiency [33,34].

Despite current research in this area, there is still no consensus on the recommended dosing or administration regime for critically ill patients. In patients with low vitamin D levels, high-dose supplementation is usually required [35,36]. There is also great variability in patient selection within critical care settings who would most benefit from supplementation. Many studies include patients with normal vitamin D values, and some studies do not even report on basal vitamin D values.

Considering the existing research gaps in this area, we aimed to analyze the available literature from randomized controlled trials on vitamin D supplementation in critically ill patients and to summarize the evidence in this narrative review.

## 2. Methods

### 2.1. Study Eligibility

Randomized controlled trials (RCTs) that included vitamin D supplementation as an intervention were eligible for inclusion. We did not set any limitations on the type of supplementation, dose of vitamin D or route of administration. We did not set any limitations for primary or secondary outcomes. We also included pilot RCTs if they reported results. In case we found both a pilot study and a full study according to that pilot, we included the full study in further analysis, and the pilot study was excluded. We also excluded studies with patients < 18 years and studies not published in English.

### 2.2. Literature Search and Selection

The literature search was conducted in October 2023. We searched MEDLINE via PubMed for RCTs using a combination of search terms: “Intensive care unit”, “Critically ill” and “Vitamin D supplementation”, as well as their corresponding MeSH terms. We used filters for interventional trials and English-language studies.

We also searched MEDLINE for published systematic reviews and meta-analyses on vitamin D supplementation in ICU patients. We searched PubMed for the terms “Intensive care unit”, “Critically ill” and “Vitamin D supplementation”. We applied filters for studies published in English, systematic reviews and meta-analyses.

In addition to MEDLINE searches, we searched the Clinicaltrials.gov registry for eligible unpublished trials in October 2023.

### 2.3. Data Extraction

From the selected studies, we extracted data on the number of patients included in the study, inclusion criteria regarding vitamin D levels, the dosage of vitamin D supplementation and the type of administration. We also analyzed the primary and secondary outcomes of the selected studies. The data were imported into a Microsoft Excel spreadsheet.

### 2.4. Statistical Analysis

Data were analyzed using descriptive statistics. Mean and median values, along with interquartile ranges (IQRs), were used where appropriate.

Due to the large heterogeneity of data—mainly different dosing regimens, types of supplementation and different outcomes—we were unable to perform a more detailed statistical analysis.

## 3. Results

### 3.1. Literature Search

The MEDLINE search for RCTs resulted in 849 results. After applying filters for RCTs, 64 results remained. A screening of titles and abstracts was performed by two authors independently for applicable studies. This resulted in 15 studies that complied with inclusion criteria.

The MEDLINE search for SRs and MAs resulted in 59 results. Two authors independently screened the titles and abstracts for eligible studies, and the screening resulted in 10 studies eligible for further analysis [28,30,37,38,39,40,41,42,43]. The authors then independently screened the references of the selected SRs and meta-analyses for RCTs, which resulted in 10 new RCTs. One of those studies was published in Chinese and was excluded from further analysis.

The search of Clinicaltrials.gov resulted in 13 results, of which one research was completed and had available results. This study was therefore included in the analysis.

Subsequently, we retrieved the full texts of 25 eligible RCTs. After independent screening by two authors, a total of 21 RCTs were finally included for further analysis. Four trials were excluded: one study reported a secondary analysis of previously published data, one study included patients <18 years, one study was a pilot study with the same outcomes as a full study, and one study employed an inappropriate intervention, as the authors used a multivitamin preparation (Vitalipid and Soluvit) for vitamin D supplementation as an intervention [44,45,46,47].

**Figure 1:**
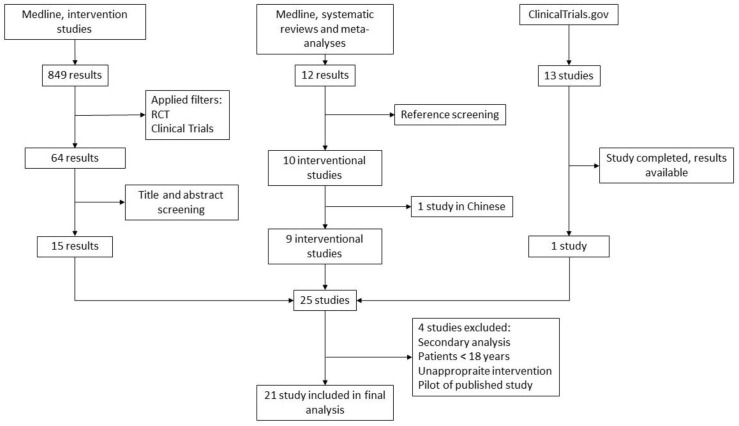
Flow diagram of the literature search. MEDLINE was searched for interventional trials and systematic reviews, and Clinical.trials.gov was searched for ongoing studies.

The flow diagram is presented in Figure 1.

### 3.2. Study Characteristics

A total of 3166 patients were included in 21 RCTs (range 24–1360) [13,46,48,49,50,51,52,53,54,55,56,57,58,59,60,61,62,63]. There was large heterogeneity in terms of patients’ characteristics and inclusion criteria. Only six studies included patients with vitamin D levels < 50 nmol/L [13,48,50,56,58,59], four studies included patients with vitamin D levels < 75 nmol/L [49,52,53,60], and two studies included patients regardless of their vitamin D levels [55,64]. In the remaining 10 studies, vitamin D levels were not mentioned as inclusion criteria. The median vitamin D levels of patients included in the analyzed studies was 37.5 nmol/L (IQR 28–47.5).

The most often used intervention was supplementation with cholecalciferol, reported in 18 studies. In 17 studies, supplementation was administered orally or via an enteral tube, in five studies, it was administered intramuscularly, and in two studies, intravenously. There was significant variability in the dosage used for supplementation. In most studies, vitamin D was administered in a bolus dose varying from 8000 IU to 600,000 IU. In studies where repeated dosing was used, a bolus dose was administered at first, followed by vitamin D administration over a variable period of days, weeks or months. In some studies, repeated doses were administered without a prior bolus dose. The median value of cumulative dose administered was 300,000 IU (IQR 200,000–510,000).

The intervention was compared to a placebo in most studies. In two studies, there was no control group reported, while in one study, the intervention group was compared to a group of patients who had normal vitamin D levels.

### 3.3. Outcomes

Regarding the increase in vitamin D levels, control values were checked in variable periods after supplementation. Sixteen studies reported an increase in vitamin D values to >50 nmol/L after intervention, while there was no increase to values >50 nmol/L reported for nine interventions. In studies that reported control values on day 7 after supplementation, there was a 66.5% (IQR 57–114) increase from the starting values, while on day 14 after the intervention, studies reported an increase of 68% (IQR 39–83) from the starting values.

One of the clinically relevant outcomes (mortality, duration of mechanical ventilation, duration of ICU stays and hospital length of stay) was reported as the main outcome in eight out of twenty-one analyzed studies. Markers of cellular immunity, inflammatory markers or vitamin D levels were reported as the main outcomes in five studies, while antioxidative capacity was reported in two studies and glucose metabolism in one study as the main outcome.

In the outcome analysis, four studies showed better mortality outcomes in the intervention groups, while most of the studies showed no difference between the intervention and control groups. Two studies reported a shorter duration of ICU stay in the intervention study, while one study reported a shorter duration of hospitalization in patients receiving vitamin D supplementation. Most studies did not report a difference in the duration of mechanical ventilation.

The list of analyzed studies and their main characteristics are presented in Table 1.

### 3.4. Data Synthesis

**Figure 2:**
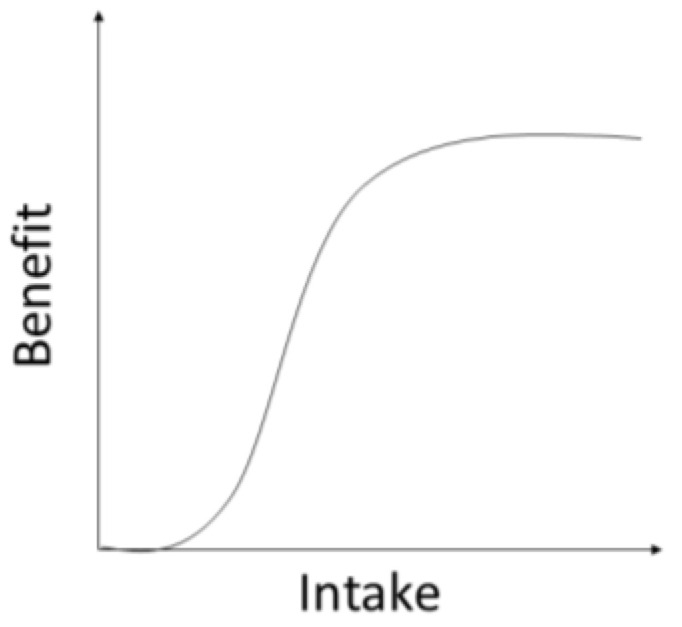
Dose-response curve for vitamin D supplementation. Supplementation in patients on the left side of the curve will lead to a steep increase in plasma concentration, while supplementation in patients on the right side of the curve will have little effect on increasing plasma concentration.

Due to different inclusion criteria, it is difficult to compare the results of supplementation effects on the selected outcomes. Some of the studies used vitamin D supplementation in patients with normal vitamin D levels, which makes it unclear what was the goal of such an intervention. Furthermore, some studies reported very low basal levels of vitamin D, and supplementation in these patients could potentially have a more significant effect compared to patients with higher basal vitamin D levels, considering the dose-response curve of vitamin D supplementation (Figure 2). Due to significant variability in supplementation protocols, as well as in the reported and analyzed outcomes, data synthesis was not possible.

## 4. Discussion

The results of our literature review indicate a large heterogeneity in the design of randomized clinical trials regarding the supplementation of vitamin D in critically ill patients. Furthermore, clinical trials show variable results in terms of different clinical outcomes. Vitamin D supplementation proved beneficial in only four of the twenty-one studies analyzed, while only two trials showed a shorter ICU stay, and one showed a shorter hospital length of stay.

Some of the important factors that can influence outcomes in clinical trials in which micronutrients include the starting levels of nutrients, as well as the dosage and route of administration of the supplement.

According to the Endocrine Society, vitamin D deficit is defined as levels <50 nmol/L [33,67], and its prevalence varies from 24 to 40% [68,69]. In critically ill patients, the prevalence is even higher, ranging from 40 to 70% [70,71]. Regarding supplementation, the dosage varies according to specific populations, ranging from 200 IU to 2000 IU/day [33,34]. There are no specific recommendations for supplementation in critically ill patients. However, there is a recommendation for the general population that the daily dose should not exceed 10,000 IU/day for prolonged periods in order to avoid toxic effects [72]. These potential side effects include a higher risk of falls, fractures and metabolic disturbances such as hypercalcemia and hyperphosphatemia. In most of the clinical studies, these doses were much higher than recommended, ranging from 50,000 to 540,000 IU. Despite such high doses, there are no reports of vitamin D intoxication other than a few cases of milder hypercalcemia that did not have any clinical effects.

The dose-response curve is presented in Figure 2. For vitamin D, the curve is sigmoid-shaped, and it is prudent to remain on the left side of the curve, in the concentration area where supplementation leads to a steep increase in concentration with a low risk of adverse effects [73]. The right side of the curve is less steep, meaning that increasing supplementation or dosage of the drug will have little effect until the ascending part of the curve is reached [73]. On the other hand, increasing the dosage in the patients who are on the right side of the curve will also have little effect on increasing serum concentrations, but it can lead to more adverse effects [73]. This non-linear response curve means that doubling the dose will not lead to a doubling of the effect in terms of increasing serum levels of vitamin D [73,74].

In studies analyzed in this review, vitamin D levels were reported in less than half of the analyzed interventional studies [13,48,49,50,52,53,56,58,59,60]. In some of the studies, both patients with normal and those with low vitamin D levels were included in the interventional groups [13,51,52,61,62,63,64]. In terms of study designs, it would be reasonable to measure vitamin D levels on admission to the ICU or before randomization of the participants, as it would be questionable to supplement vitamin D to patients with normal vitamin D levels or to those whose vitamin D levels are unknown. Furthermore, since several studies did not report basic levels of vitamin D [51,58], it is difficult to compare the effects of supplementation.

Another problem identified in this literature review is the large number of supplementation protocols, with varying doses, dosing intervals and routes of administration. According to current guidelines and recommendations, the maximal daily dose of vitamin D supplementation considered safe is 10,000 IU, and high bolus doses of >100,000 IU are not recommended [74]. Despite numerous studies, there is no agreement on the most efficient dosing regimen for vitamin D supplementation. High enteral or parenteral doses did not show beneficial effects on clinical outcomes in a number of studies [29,50,75,76,77]. On the other hand, there are studies showing potential beneficial effects of everyday vitamin D supplementation [78]. In a systematic review by Pal et al., the authors showed the beneficial effect of vitamin D supplementation in COVID-19 patients [26]. The cumulative dose of orally administered cholecalciferol varied from 80,000 IU to 400,000 IU. There was no difference in outcomes when comparing the administration of low (less than 200,000 IU) versus high cumulative doses [26].

Due to the short half-life of vitamin D (12–24 h), even high bolus doses of up to 100,000 IU are removed from circulation within a week of administration [77,79,80]. Since vitamin D is lipid-soluble, it was speculated that after a high bolus dose, a proportion of vitamin D would be stored in fat tissue. However, this theory was not proven correct, as there were no measurable concentrations of supplemented vitamin D once it was removed from circulation [81,82]. In this review, the median weekly cumulative dose of vitamin D was 300,000 IU (IQR 200,000–510,000 IU). Despite these high cumulative doses, in nine studies (41%), vitamin D levels did not increase to levels >50 nmol/L during the study period. Based on these data, it would be sensible to use everyday supplementation in order to achieve stable serum concentrations of vitamin D [83,84]. Furthermore, this type of supplementation could potentially lead to increased availability of vitamin D metabolites, which might lead to more beneficial effects on clinical outcomes [79].

Although vitamin D supplementation has been shown to be beneficial in the prevention and mitigation of acute respiratory illness [78,85], its beneficial effect on clinical outcomes in critically ill patients has not yet been determined [26,29,50,75,76,86]. Lan et al. showed better clinical outcomes in patients receiving vitamin D supplementation; however, the difference was not statistically significant [28]. Based on a meta-analysis of nine RCTs with 1867 critically ill adults, Lan et al. reported no significant differences in 28-day mortality, ICU and hospital LOS, or duration of mechanical ventilation. Since the clinical characteristics of critically ill patients were heterogeneous and the criteria for vitamin D deficiency were not uniform across the included RCTs, the authors suggested that diverse patient populations may have obscured potential positive benefits of vitamin D supplementation [28]. Similar results were shown in a systematic review that analyzed the effect of vitamin D supplementation in COVID-19 patients [87].

In this literature review, we did not use the methodology for systematic reviews. Due to the large heterogeneity in study designs and supplementation protocols, we were unable to conduct any quantitative analysis of the data. Furthermore, it is possible that some publications were missed during the literature search and, therefore, were not included in this review.

## 5. Conclusions

There is great variability in trial designs regarding the selection of patients, dosage, dosing intervals and routes of administration of vitamin D supplements. Furthermore, there is a large heterogeneity in the reporting of clinically relevant outcomes. A significant proportion of RCTs did not report basic vitamin D levels, or they included patients with both low and normal vitamin D levels. Regarding the results of the analyzed trials, only a few showed beneficial effects of vitamin D supplementation on clinical outcomes. These effects were associated with neither the dosage nor dosing regimens used as intervention.

It is possible that vitamin D supplementation could have beneficial effects on clinical outcomes in patients with milder clinical presentations of respiratory disease. In critically ill patients, it is likely that vitamin D supplementation, as a single intervention, cannot influence clinical outcomes regardless of the dosing regimens.

Better study designs are mandatory for future clinical research regarding vitamin D supplementation. The patient population should be more homogenous regarding their clinical characteristics, and measurements of basal vitamin D values should be mandatory prior to patient inclusion in the study, as there is little benefit in supplementing patients with normal vitamin D levels. Furthermore, supplementation protocols should be standardized, as there is currently great variability regarding the route of administration, total dose of vitamin D as well as administration intervals. Emphasis should also be on study outcomes, as there is significant variability in reported outcomes, especially on clinically relevant outcomes. All these factors limit the possibility of data synthesis.

## Figures and Tables

**Table 1:** Studies included in analysis.

Author, Year	N Participants Included in Study	N Participants Included in Analysis	Participants	Vitamin D Status as Inclusion Criteria	Mean Vitamin D Values (nmol/L)	Intervention	Comparator	Main Outcome	Other Outcomes	Signifficance of the Outcomes
Ginde, 2019 [48]	1360	1059	Adult ICU patients (33.7% mechanically ventilated)	<50 nmol/L	Intervention: 28 ± 12; Control: 27 ± 12	Cholecalciferol, EN; 1 × 540,000 IU	Placebo	90-day mortality	Hospital LoS; Ventilator free days	No significant difference in primary or secondary outcomes
Bychinin, 2022 [49]	110	56	Adult patients, COVID-19 (33.6% mechanically ventilated)	<75 nmol/L	Intervention: 24 (IQR 14–52.5); Control: 27.5 (IQR 21.5–37.5)	Cholecalciferol, EN; 60,000 IU weekly + 5000 IU daily/5 weeks	Placebo	Cell immunity and inflammatory markers on the 7th day after the intervention	Mortality; ICU LoS; Hospital LoS; Duration of respiratory support	Higher values of NK and NKT cells in the intervention group; Shorter ICU and hospital Los and lower number of days on mechanical ventilation in the control group
Hasanloei, 2019 [13]	80	72	Adult trauma patients, mechanically ventilated	25–75 nmol/L	Intervention: 42.7 ± 11.3; 46.6 ± 8.2; Control: 42.5 ± 8.1	Cholecalciferol; 50,000 IU PO/6 days 300,000 IU IM	Placebo	Duration of respiratory support	Mortality; ICU LoS	Less days on respiratory support in both intervention groups; No difference in mortality and shorter ICU LoS in both intervention groups
Amrein, 2014 [50]	492	475	Adult ICU patients (64% mechanically ventilated)	<50 nmol/L	Intervention: 32.5 ± 10; Control: 32.7 ± 10.7	Cholecalciferol, EN; 540,000 IU bolus + 90,000 IU monthly/5 months	Placebo	Hospital LoS	Hospital mortality; 6-months mortality; ICU LoS; Hospital LoS; Duration of respiratory support	No significant difference in primary or secondary outcomes
Leaf, 2014 [51]	67	67	Adult patients with sepsis (70% mechanically ventilated)	Not measured	Not reported	Calcitriol, IV; 2 mcg	Placebo	Immune and inflammatory markers	28-day mortality; ICU LoS; Hospital LoS; Duration of respiratory support	No significant difference in primary or secondary outcomes
Miroliaee, 2017 [52]	51	46	Adult patients with VAP	<75 nmol/L	Intervention: 42.8 ± 15.3; Control: 48.7 ± 11.5	Cholecalciferol, IM; 300,000 IU	Placebo	Inflammatory markers on day 7	28-day mortality	Lower IL-6 levels in the intervention group; no difference in CRP levels and lower 28-day mortality in the intervention group
Miri, 2018 [53]	40	40	Adult ICU patients, mechanically ventilated	<70 nmol/L	Intervention: 21.1 ± 17; Control: 28.4 ± 45.6	Cholecalciferol, IM; 300,000 IU	Placebo	Duration of respiratory support	28-day mortality; ICU LoS; Hospital LoS	No difference in the primary outcome; lower mortality in intervention group and no difference in ICU LoS
Bhattacharyya, 2021 [54]	63	63	Adult patients with sepsis	Not reported	Intervention 30.1 ± 15.5; Control: 38.7 ± 26.5	Cholecalciferol, EN; 540,000 IU	Placebo	ICU LoS	90-day mortality; Hospital LoS; Duration of respiratory support	No significant difference in primary or secondary outcomes
Yousefian, 2019 [55]	99	99	Adult patients with CVI, mechanically ventilated	Patients with normal and those with low vitamin D levels	Intervention: 22.1 ± 5.7; 26.3 ± 12; Control: 70 ± 25	Cholecalciferol, EN; 300,000 IU 3× weekly + calcium 500 mg	Placebo, patients with vitamin D > 50 nmol/L	Duration of respiratory support		Shorter duration of respiratory support in the control group
Dickerson, 2015 [56]	65	65	Adult trauma patients	<50 nmol/L	Intervention: 36 ± 6 40 ± 7 37 ± 6	Ergocalciferol, EN; 50,000 IU 1×, 2× or 3× weekly	/	Vitamin D levels after two weeks of supplementation	ICU LoS;	Higher number of patients reached normal vitamin D levels in group 3
Sistanizad, 2021 [57]	36	30	Adult patients, mechanically ventilated	Not reported	Intervention: 18 ± 5.8; Control: 13.1 ± 1.8	Cholecalciferol, IM; 300,000 IU	Placebo	Antioxidative capacity	28-day mortality; ICU LoS; Duration of respiratory support	Increased antioxidative capacity in the intervention group; lower mortality and ICU Los and respiratory support in the intervention group
Karsy, 2022 [58]	274	267	Adult patients, head trauma, vitamin D deficiency	<50 nmol/L	/	Cholecalciferol, EN; 540,000 IU	Placebo	Hospital LoS	6-month mortality; ICU LoS	No significant difference in primary or secondary outcomes
Leaf, 2022 [51]	150	150	Adult patients, High risk of AKI (83% mechanically ventilated)	Not reported	Intervention: 47 (IQR 28–65) 43.2 (IQR 26.2–71) Control: 36.2 (IQR 24.2–57.5)	Calcifediol, PO, 400 mcg × 1 + 200 mcg/4 days; Calcitriol, PO, 4 mcg/5 days	Placebo	7-day mortality; RRT after 7 days	28-day mortality; ICU LoS; Hospital LoS	No significant difference in primary or secondary outcomes
Amrein, 2011 [59]	25	25	Adult patients, vitamin D deficiency (84% mechanically ventilated)	<50 nmol/L	Intervention: 32.7; Control: 32.2	Cholecalciferol, EN; 540,000 IU	Placebo	Number of patients with vitamin D levels > 50 nmol/L within 7 days	28-day mortality; ICU LoS; Hospital LoS; Duration of respiratory support	Higher vitamin D levels in the intervention group; no difference in secondary outcomes
Alizadeh, 2016 [60]	59	50	Adult surgical patients with glucose intolerance and low vitamin D levels	<75 nmol/L	Intervention: 30.2 ± 12.8; Control: 27 ± 15.7	Cholecalciferol, IM; 600,000 IU	Placebo	Blood glucose levels and metabolic markers on day 7		Higher levels of adiponectin in the intervention group and no difference in other outcomes
Quraishi, 2015 [61]	30	30	Adult patients with sepsis	Not reported	Intervention: 37.5 (IQR 30–50); 42.5 (IQR 32.5–62.5); Control: 47.5 (IQR 32.5–55)	Cholecalciferol, EN; 200,000 IU 400,000 IU	Placebo	Vitamin D levels on the 3rd, 5th and 7th day after the intervention	30-day mortality; ICU LoS; Hospital LoS	Higher levels of vitamin D in both intervention groups; shorter hospital LoS for both intervention groups and no difference in ICU LoS and mortality
Smith, 2018 [62]	30	30	Adult patients, mechanically ventilated	Not reported	Intervention: 58 ± 19.5 50 ± 18.2 Control: 53.75 ± 30.5	Cholecalciferol, EN; 50,000 IU/5 days (total 250,000 IU) 100,000 IU/5 days (total 500,000 IU)	Placebo	Hepcidin and hemoglobin levels 4 weeks after the intervention		Higher levels of haemoglobin and lower levels of hepcidin in the second intervention group
Han, 2018 [65]	30	30	Adult patients, mechanically ventilated	Not reported	Intervention: 58 ± 19.5 50 ± 18.25 Control: 53.75 ± 30.5	Cholecalciferol, EN; 50,000 IU/5 days (total 250,000 IU) 100,000 IU/5 days (total 500,000 IU)	Placebo	Antioxidative capacity		Decreased levels of GSSG in intervention group and no difference in other outcomes
Ingels, 2020 [66]	24	24	Adult patients, ICU LoS > 10 dana	Not reported	Intervention: 23 (IQR 18–32.7); Control: 17 (IQR 12.7–25.5)	Cholecalciferol, IV; 200 mcg bolus + 15 mcg daily/10 days	Placebo	Inflammatory markers and bone metabolism markers		No significant difference in the reported outcomes
Nair, 2015 [64]	50	50	Adult patients, SIRS	Regardless of vitamin D levels	Intervention: 52 (IQR 40–67) 42 (IQR 32–62)	Cholecalciferol, IM; 150,000 IU 300,000 IU	No control group	Vitamin D levels increase on days 1, 7 and 14	14-day mortality; ICU LoS; Hospital LoS	Increase in vitamin D levels in the intervention group; no difference in secondary outcomes
Han, 2016 [63]	31	30	Adult patients, duration of mechanical ventilation > 72 h, ICU LoS > 96 h	Not reported	Intervention: 58 ± 19.5 50 ± 18.2 Control: 53.7 ± 30.5	Cholecalciferol, EN; 50,000 IU/5 days (total 250,000 IU) 100,000 IU/5 days (total 500,000 IU)	Placebo	Free vitamin D levels on day 7 after the intervention		No significant difference in reported outcomes
